# Copper Oxide Nanoparticles Stimulate the Immune Response and Decrease Antioxidant Defense in Mice After Six-Week Inhalation

**DOI:** 10.3389/fimmu.2022.874253

**Published:** 2022-04-25

**Authors:** Jana Tulinska, Miroslava Lehotska Mikusova, Aurelia Liskova, Milena Busova, Vlasta Masanova, Iveta Uhnakova, Eva Rollerova, Radka Alacova, Zora Krivosikova, Ladislava Wsolova, Maria Dusinska, Mira Horvathova, Michaela Szabova, Norbert Lukan, Martina Stuchlikova, Daniel Kuba, Zbynek Vecera, Pavel Coufalik, Kamil Krumal, Lukas Alexa, Lucie Vrlikova, Marcela Buchtova, Jana Dumkova, Pavel Piler, Vojtech Thon, Pavel Mikuska

**Affiliations:** ^1^ Faculty of Medicine, Slovak Medical University, Bratislava, Slovakia; ^2^ Institute of Hygiene and Epidemiology, First Faculty of Medicine, Charles University and General University Hospital in Prague, Prague, Czechia; ^3^ Faculty of Public Health, Slovak Medical University, Bratislava, Slovakia; ^4^ Health Effects Laboratory, Norwegian Institute for Air Research, Kjeller, Norway; ^5^ National Transplant Organization, Bratislava, Slovakia; ^6^ Department of Environmental Analytical Chemistry, Institute of Analytical Chemistry of the Czech Academy of Sciences, Brno, Czechia; ^7^ Laboratory of Molecular Morphogenesis, Institute of Animal Physiology and Genetics, Czech Academy of Sciences, Brno, Czechia; ^8^ Department of Histology and Embryology, Faculty of Medicine, Masaryk University, Brno, Czechia; ^9^ RECETOX, Faculty of Science, Masaryk University, Brno, Czechia

**Keywords:** copper oxide nanoparticles, immunotoxicity, immune response, lymphocytes, cytokines, inflammation, phagocytic activity and respiratory burst, antioxidant defense

## Abstract

Copper oxide nanoparticles (CuO NPs) are increasingly used in various industry sectors. Moreover, medical application of CuO NPs as antimicrobials also contributes to human exposure. Their toxicity, including toxicity to the immune system and blood, raises concerns, while information on their immunotoxicity is still very limited. The aim of our work was to evaluate the effects of CuO NPs (number concentration 1.40×10^6^ particles/cm^3^, geometric mean diameter 20.4 nm) on immune/inflammatory response and antioxidant defense in mice exposed to 32.5 µg CuO/m^3^ continuously for 6 weeks. After six weeks of CuO NP inhalation, the content of copper in lungs and liver was significantly increased, while in kidneys, spleen, brain, and blood it was similar in exposed and control mice. Inhalation of CuO NPs caused a significant increase in proliferative response of T-lymphocytes after mitogenic stimulation and basal proliferative activity of splenocytes. CuO NPs significantly induced the production of IL-12p70, Th1-cytokine IFN-γ and Th2-cytokines IL-4, IL-5. Levels of TNF-α and IL-6 remained unchanged. Immune assays showed significantly suppressed phagocytic activity of granulocytes and slightly decreased respiratory burst. No significant differences in phagocytosis of monocytes were recorded. The percentage of CD3^+^, CD3^+^CD4^+^, CD3^+^CD8^+^, and CD3^-^CD19^+^ cell subsets in spleen, thymus, and lymph nodes did not differ between exposed and control animals. No changes in hematological parameters were found between the CuO NP exposed and control groups. The overall antioxidant protection status of the organism was expressed by evaluation of GSH and GSSG concentrations in blood samples. The experimental group exposed to CuO NPs showed a significant decrease in GSH concentration in comparison to the control group. In summary, our results indicate that sub-chronic inhalation of CuO NPs can cause undesired modulation of the immune response. Stimulation of adaptive immunity was indicated by activation of proliferation and secretion functions of lymphocytes. CuO NPs elicited pro-activation state of Th1 and Th2 lymphocytes in exposed mice. Innate immunity was affected by impaired phagocytic activity of granulocytes. Reduced glutathione was significantly decreased in mice exposed to CuO NPs.

## 1 Introduction

Demand for copper oxide nanoparticles (CuO NPs) has grown tremendously, driven by penetration in electrical engineering and electronics, paints and coatings, semiconductors, energy storage, catalysts, and other fields. Nano-copper oxide is a widely used material that leads to improved performance of end products due to its exceptional physicochemical properties (1). In addition, CuO NPs have shown their potential in pharmaceutical and biomedical applications, such as antibacterial, antifungal, anticancer, and drug delivery agents (2, 3). Moreover, they can be used as cleansing agents for heavy metals from wastewater (4, 5). The application of CuO NPs to soils, as fertilizers, fungicides, or pesticides, has been proposed to improve the sustainability of agriculture (6, 7).

Copper (Cu) is a trace element that is essential for a variety of biological processes. It is required for energy transformation; iron metabolism; the production of hemoglobin, melanin, myelin, collagen, and elastin; the synthesis of hormones; and defense against oxidative damage (8, 9). However, Cu can be toxic when present in excess, and it is associated with the pathogenesis of several diseases (10).

The expanding production and use of CuO NPs have raised concerns about the potential for undesirable human health and environmental effects. Inhalation is the main route of entry of NPs into the body (11). CuO NPs are prone to diffusion in the ambient air as aerosols and are retained in the lungs for a long time after inhalation (12). NPs interact with the lung epithelium leading to inflammation. As the size of NPs decreases, the disposition rate of NPs in lungs significantly increases. From the lungs, the NPs are transported to other regions of body through the blood circulatory system, accumulating in the various body organs and causing toxic effects at the different sites (11).

NPs tend to exhibit quite different toxicological effects compared with larger particles of the same chemical composition (13). The key factors that influence the toxicity of CuO NPs are size, shape, surface modification, morphology, and concentration (11). The systemic distribution of particles depends on the ability to diffuse through tissues or cells, which is partly determined by the surface charge of the particles. Once inside the cells, NPs may interact with organelles and generate reactive oxygen species (ROS) which disrupt normal cellular functions (14). ROS induce oxidative stress, cause lipid peroxidation, and damage defense mechanisms of cells by depletion of reduced glutathione (tripeptide γ-glutamyl-cysteinyl-glycine, GSH). In addition, ROS increase superoxide dismutase and catalase activities in cells (15).

*In vitro* studies on exposure of human cells to CuO NPs have shown high cytotoxicity and ability to cause DNA damage, induction of oxidative stress, and cell death (16–20). Compared with TiO_2_, ZnO, Fe_2_O_3_, Fe_3_O_4_ and CuZnFe_2_O_4_ NPs, copper oxide NPs are the most potent regarding cytotoxicity and DNA damage. CuO NPs have also been found to be much more toxic than Cu ions. NPs may serve as “Trojan-horse type carriers” enabling the transport of metal ions into the cells. While cell membranes are good barriers for most ions, toxic Cu ions can be released inside cells as the small size of CuO NPs allows them to enter the cell (21).

Results from *in vivo* studies indicate that oral administration of CuO NPs induces oxidative stress, enhanced ROS generation, inflammation, apoptosis, and histopathological alterations in various organs, such as liver, kidney, stomach, and bone marrow (22–25). In mice exposed to NPs *via* inhalation, CuO NPs induced pulmonary inflammation, apoptosis, generation of ROS, and fibrosis (12). In rats, cytotoxicity and pulmonary inflammation and toxicity response have also been observed after CuO NP inhalation (26, 27). Pietrofesa et al. (28) reported that inhalation of CuO NPs elicits an inflammatory response resulting in damage to cells and lung tissues. There are few studies on the effects of CuO NP inhalation on the immune system. Holan et al. (29) observed that the inhalation of CuO NPs in mice significantly alters the composition of cell populations of innate immunity and modulates the proliferation and production of cytokines by cells of the adaptive immune system. Ilves et al. (30) found that inhalation exposure to CuO NPs exacerbates allergic airway inflammation by causing neutrophilia in the mouse lungs. Rossner et al. (31) observed the impacts on immune response, cell cycle regulation, cell adhesion and apoptosis, as well as pathways potentially implicated in carcinogenesis in a study focused on transcriptomic and epigenetic changes in mice following CuO NPs inhalation.

Several studies have demonstrated that immune-mediated inflammation and oxidative stress play an important role in the pathogenesis of NP-induced injury. Published data on systemic immune response and function of immune cells upon inhalation administration of CuO NPs are very limited. The aim of our study was to investigate the effects of CuO NPs on immune response and antioxidant defense in mice after six-weeks of inhalation. Our study brings new knowledge on the effects of CuO NPs on antioxidative status, inflammatory response, and function of cells of the natural and adaptive immune response in exposed mice.

## 2 Material and Methods

### 2.1 Animals

Adult female mice (ICR line, 6-week-old, average body weight 24 g) were obtained from Masaryk University (Brno, Czech Republic). Before the experiment, the mice were acclimated to laboratory conditions for a period of one week. Commercial diet and water were provided ad libitum.

All animal experiments were carried out in accordance with the Guidelines for Care and Use of Laboratory Animals of Institute of Analytical Chemistry of the Czech Academy of Sciences (the Ministry of Agriculture of the Czech Republic, No. 30286/2016-MZE -17214) and approved by the Animal Ethics Committee of Institute of Analytical Chemistry of the Czech Academy of Sciences (55/2015).

### 2.2 Preparation of NPs

CuO NPs were generated continuously *in situ* before inhalation inside the ceramic reactor tube of a vertically oriented furnace (Carbolite, Hope Valley, UK) using a thermal decomposition of metal organic precursor copper(II) acetylacetonate (Aldrich, Milwaukee, WI, USA) and subsequent oxidation at a temperature of 700°C. The vapors of copper(II) acetylacetonate were generated from a solid form of copper(II) acetylacetonate in a saturator at 136°C and released vapors were transported by nitrogen (purity 99.999%, at a flow rate of 0.810 L/min) into the reactor, where the copper(II) acetylacetonate was oxidized in the presence of oxygen (99.99%, 0.33 L/min), added by means of a silica fused capillary (30 cm) inserted into a ceramic reactor tube. At the furnace output, CuO NPs were diluted with a U-HEPA filtrated air stream at a flow rate of 2.11 L/min, and then split into two streams at a ratio of 1:1. Prior to entering the inhalation chamber, both streams were further diluted with a stream of purified humidified air (15 L/min) and used for whole-body inhalation experiments.

**Figure 1:**
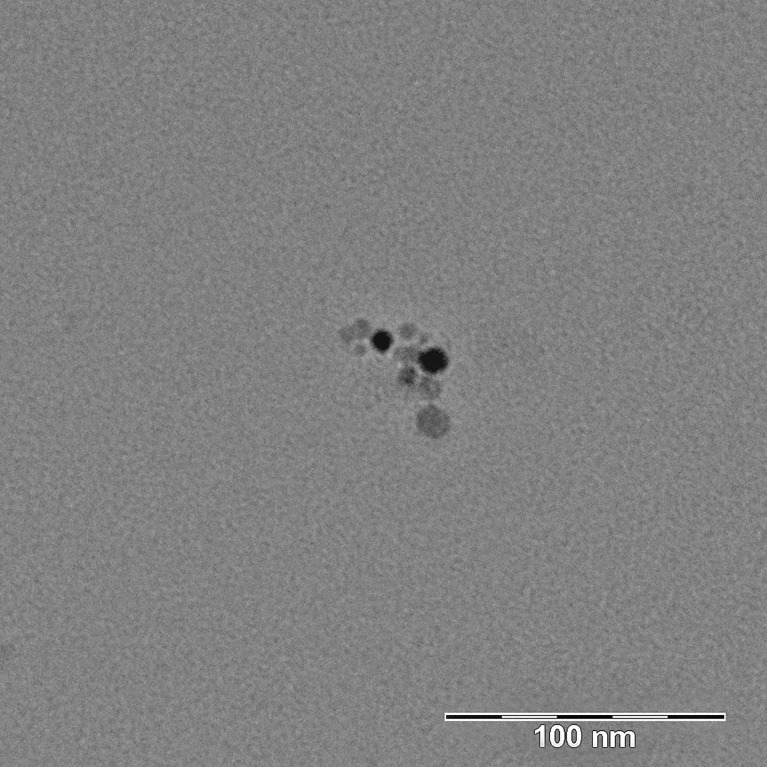
Transmission electron micrograph of CuO NPs placed on the TEM grid. CuO NPs were measured in air inside the inhalation cage by Scanning Mobility Particle Sizer. They were composed of agglomerates of primary particles with a diameter of 3-11 nm.

The size and shape of generated CuO NPs were characterized by electron microscopy (EM). CuO NPs were collected by electrostatic precipitation using a nanometer aerosol sampler (model 3089, TSI) on EM grids (copper S160-4, 3 mm in diameter, 400 mesh grids, Agar Scientific, Electron Technology, Stansted, Essex, UK). The samples were examined using Morgagni™ 268 TEM (FEI Company, Eindhoven, Netherlands), working at 90 kV and equipped with a *Veleta CCD camera* (Olympus, Münster, Germany). The designated structures were measured using iTEM software. The micrograph (**Figure 1**) showed that the CuO NPs in air inside the inhalation cage, measured by Scanning Mobility Particle Sizer (model 3972L, TSI, USA), were composed of agglomerates of primary particles with a diameter of 3-11 nm.

The surface area of the generated CuO NPs was 4.09 × 10^3^ μm^2^/cm^3^. Surface area was calculated from data of NP size distribution using the SMPS software (32).

### 2.3 Exposure to CuO NPs

Inhalation exposure to CuO NPs was conducted in a special inhalation chamber (33). The inhalation chamber was made of glass and stainless steel and contained four stainless steel inhalation cages. A control group of mice was also placed into the inhalation chamber in a cage without the exposure to NPs.

**Figure 2:**
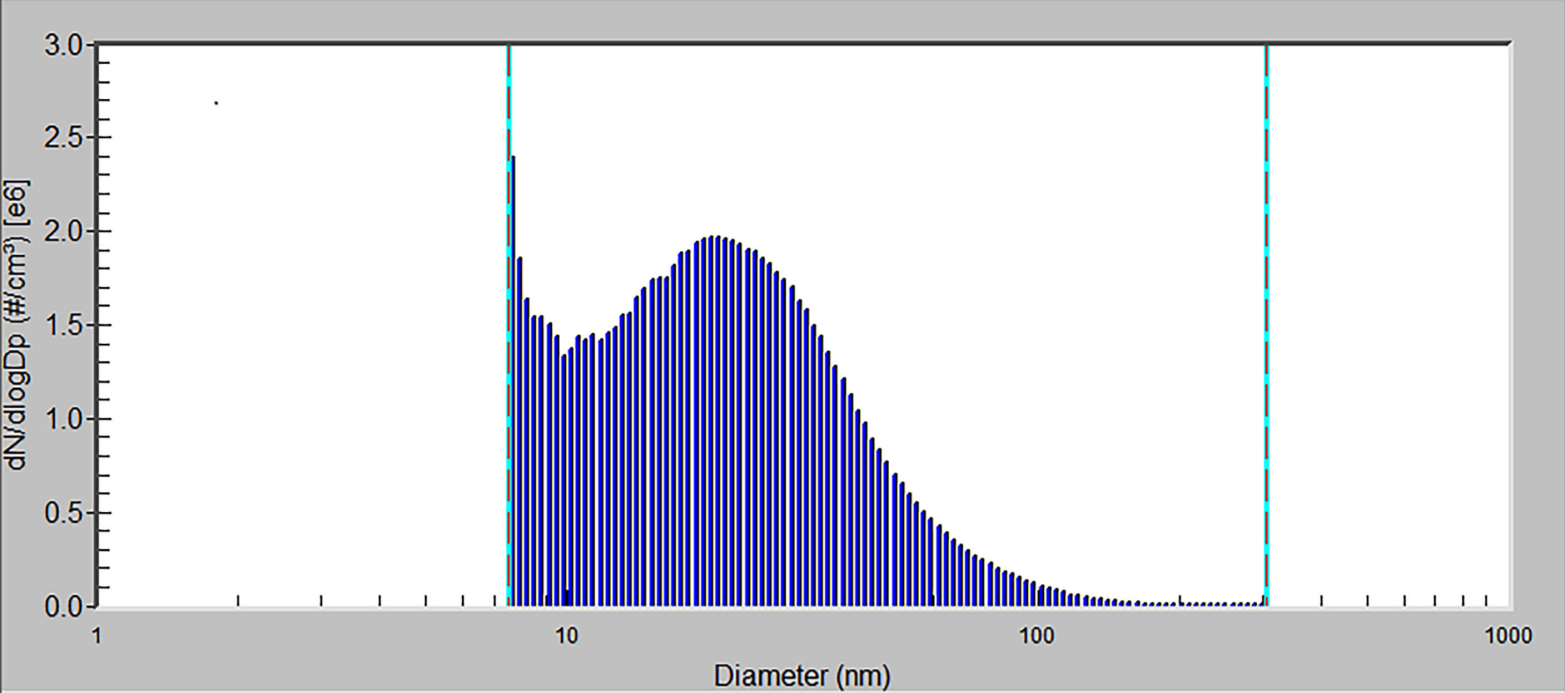
The size distribution of CuO NPs by number. x-axis – particle diameter (a logarithmic scale), y-axis – number concentration of particles (the number concentration is normalized by the size range of particles) by number.

An air-conditioning system maintained air passing through the inhalation cages at constant temperature, relative humidity, and pressure. The air parameters were measured and recorded on-line in 1-min periods. Animals were maintained on a standard 12-h light/dark cycle. The behavior and health condition of the mice were continuously monitored by a camera system. The distributions of generated NPs were determined directly in the inhalation cages using a Scanning Mobility Particle Sizer. Size distribution is shown in **Figure 2**. A special feeding device (a tube closed at the top with feed falling down to a feeder) has been constructed to minimize oral intake of NPs from possible contamination of commercial diet granules due to adsorption of CuO NPs on their surface.

Mice were exposed to the CuO NPs continuously for 6 weeks, 24 h/day, 7 days/week. Control animals were exposed to the same air as the experimental groups, just without the NP supplement. The number concentration of CuO NPs was 1.40 × 10^6^ particles/cm^3^ (mode 7.64 nm, geometric mean diameter 20.4 nm, geometric standard deviation 1.83). Average mass concentration of CuO NPs was 32.5 µg CuO/m^3^. Estimated total deposited dose over the 6-week inhalation period was 0.20 µg of CuO per gram of mouse body weight. At the end of the inhalation experiment, mice were directly decapitated and immediately dissected.

### 2.4 Determination of Cu Content in Mouse Organs and Blood

In total, 5 control and 5 exposed mice were studied, from which lung, brain, spleen, liver and kidney and blood samples were taken for analysis. The determination of Cu in organs and blood samples was performed in clean room laboratories. The whole organs and blood were decomposed in 3 mL of concentrated sub-boil grade (quartz distillation system model MSBQ 2, Maasen, Eningen, Germany) nitric acid by microwave assisted digestion. The samples were treated in pre-cleaned quartz tubes of a closed pressurized autoclave system (UltraWave, Milestone, Italy). The decomposition program consisted of four steps: 1^st^ step – 10 min with a temperature ramp between 100 and 120°C, 2^nd^ step – 5 min with a temperature ramp between 120 and 200°C, 3^rd^ step – 3 min with a temperature ramp between 200 and 250°C, 4^th^ step – 5 min at 250°C. After cooling down (for approx. 10 min), digests were quantitatively transferred to HDPE vials, diluted, and adjusted with ultrapure water (Ultra Clear system, SB Barsbüttel, Germany) to the final mass of 11 g for organs and 3 g for blood. Ten blank samples were similarly processed in parallel. The content of Cu in digests was determined by electrothermal atomic absorption spectrometry (ET-AAS) employing AAnalyst 600 Perkin Elmer (USA) instrumentation under recommended conditions. A method of standard addition was applied for the quantitation.

### 2.5 Immune Assays

#### 2.5.1 Phenotypic Analysis of Spleen, Thymus, and Lymph Nodes

The spleen, abdominal lymph nodes, and thymus were placed in complete RPMI 1640 culture medium (Sigma, NJ, USA). Details of the procedure are described in Tulinska et al. (34). The following antibodies were purchased from eBioscience (MA, USA) and used to stain the cells: Anti-Mouse CD3e PE, Anti-Mouse CD4 FITC, Anti-Mouse CD8a PerCP-eFluor^®^ 710, Anti-Mouse CD19 FITC and Anti-Mouse CD335 PerCP-eFluor^®^ 710, isotypic controls (IK): Armenian Hamster IgG IK PE, Rat IgG2aƙ IK FITC, Rat IgG2aƙ IK PerCP-eFluor^®^ 710. Concentrations recommended by the manufacturer were applied. The Cytomics FC500 flow cytometer (Beckman Coulter, CA, USA) was used for sample analysis. The percentages of CD3^+^, CD3^+^CD4^+^, CD3^+^CD8^+^ in spleen, thymus, and lymph nodes, and CD3^-^CD335^+^, CD3^-^CD19^+^ cells in spleen and lymph nodes, were examined in duplicate samples.

#### 2.5.2 *In Vitro* Lymphocyte Proliferation Assay

Single-cell lymphocyte populations were prepared from the spleen and lymph nodes of the mice as described previously (34). Cell suspensions were cultured without mitogens (non-stimulated cultures), and with mitogens concanavalin A (Con A; 2.5 µg/mL), phytohemagglutinin (PHA; 25 µg/mL), and pokeweed mitogen (PWM; 2.5 µg/mL) (all Sigma, NJ, USA). Cells were incubated for 48 h at 37°C and 5% CO_2_. Thereafter, cultures were pulsed with 1 μCi [^3^H]-thymidine (MGP, KA, USA) and incubated for another 24 h. The cell cultures were harvested on glass filter papers and placed in scintillation fluid (Perkin Elmer, MA, USA). Radioactivity was measured using a Beta Scintillation counter Microbeta 2 (Perkin Elmer, MA, USA). Counts per minute (cpm)/cell culture were determined in triplicate for each variable.

#### 2.5.3 *In Vitro* Production of Cytokines

After culturing spleen cells with mitogen Con A in microtitrate plates at 37°C for 48 h, supernatants were stored at -70°C. The Essential Th1/Th2 Cytokine 6-Plex Mouse ProcartaPlex™ Panel (eBioscience, MA, USA) was used to measure the levels of IL-4, IL-5, IL-6, Interferon-γ (IFN-γ), IL-12p70, and Tumor necrosis factor-α (TNF-α), following the instructions of the manufacturer. Values extrapolated beyond the calibration curve (cc) as well as values out of range of cc (below, above) were discarded.

#### 2.5.4 Phagocytic Activity of Granulocytes and Monocytes and Respiratory Burst of Phagocytes

The details of the assay were published previously (34). In brief, mouse heparinized whole blood was mixed with hydroethidine. Subsequently, samples were incubated with fluorescein-labelled *Staphylococcus aureus* (SA, Invitrogen, MA, USA) at 37°C. They were then put on ice and cold lysis solution was added. In the case of the control tubes, SA bacteria were added after the lysis solution. The percentage of phagocytic monocytes, percentage of phagocytic granulocytes, and percentage of granulocytes with respiratory burst were measured in duplicate samples using Cytomics FC500 flow cytometer (Beckman Coulter, CA, USA). The results were assessed by flow cytometry as follows: % of phagocytic granulocytes = phagocytic granulocytes/all granulocytes.

### 2.6 Hematological Analysis

Blood samples were collected using tubes with anticoagulant (EDTA) and mixed gently. Hematological analysis was performed using a hematological analyzer Sysmex K-4500 (SYSMEX TOA Medical Electronics Co. LTD, Japan).

### 2.7 Antioxidant Status, Reduced Glutathione, and Oxidized Glutathione

Concentrations of reduced (GSH) and oxidized (GSSG) glutathione, as markers of oxidative stress and antioxidant defense of the organism, were determined with the Bioxytech^®^ GSH/GSSG-412™ assay kit (Oxis International, Inc.) based on the method of Ellman (35), which was modified by Tietze (36). Immediately after blood collection into the plastic tubes with EDTA as anticoagulant, 100 μL of each whole blood sample for GSSG determination was transferred into 1.5 mL (Eppendorf) microtube with 10 μL of the scavenger M2VP (1-methyl-2-vinyl-pyridium-trifluoromethane sulfonate) to prevent oxidation of GSH to GSSG during sample preparation. Next, 50 μL of whole blood sample for GSH determination was transferred into 1.5 mL microtube (Eppendorf) without treatment. All samples were frozen at -80°C until the analysis. Immediately before analysis, blood samples were thawed and mixed. After all procedures, and reaction with Ellman’s reagent (5,5´-dithiobis-2-nitrobenzoic acid, DTNB), the samples were measured by a spectrophotometric reader at 412 nm (Epoch, BioTek) in duplicates. Concentrations of GSH and GSSG are expressed in μmol/L. The GSH/GSSG ratio was calculated using the assay kit instructions.

### 2.8 Statistical Analysis

Statistical analysis was performed using SPSS 23.0 software. Data were reported as means ± standard error of the mean (SEM). The multiple measurements from each individual were averaged and used as a single value for analysis. The Shapiro-Wilk’s test was applied to examine the normality of data distribution. Data on proliferative activity of lymphocytes and oxidative stress (GSH, GSSG) were tested for identification and elimination of outliers (Grubbs’ test). A Student T-test (for normally distributed datasets) or Mann–Whitney tests (for non-normally distributed datasets) were used to determine differences between the exposed and control groups. Differences with p < 0.05 were considered statistically significant. P values are reported as follows: * p < 0.05; ** p < 0.01; *** p < 0.001.

## 3 Results

### 3.1 CuO NP Inhalation Increased Cu Content in the Lungs and Liver

**Figure 3:**
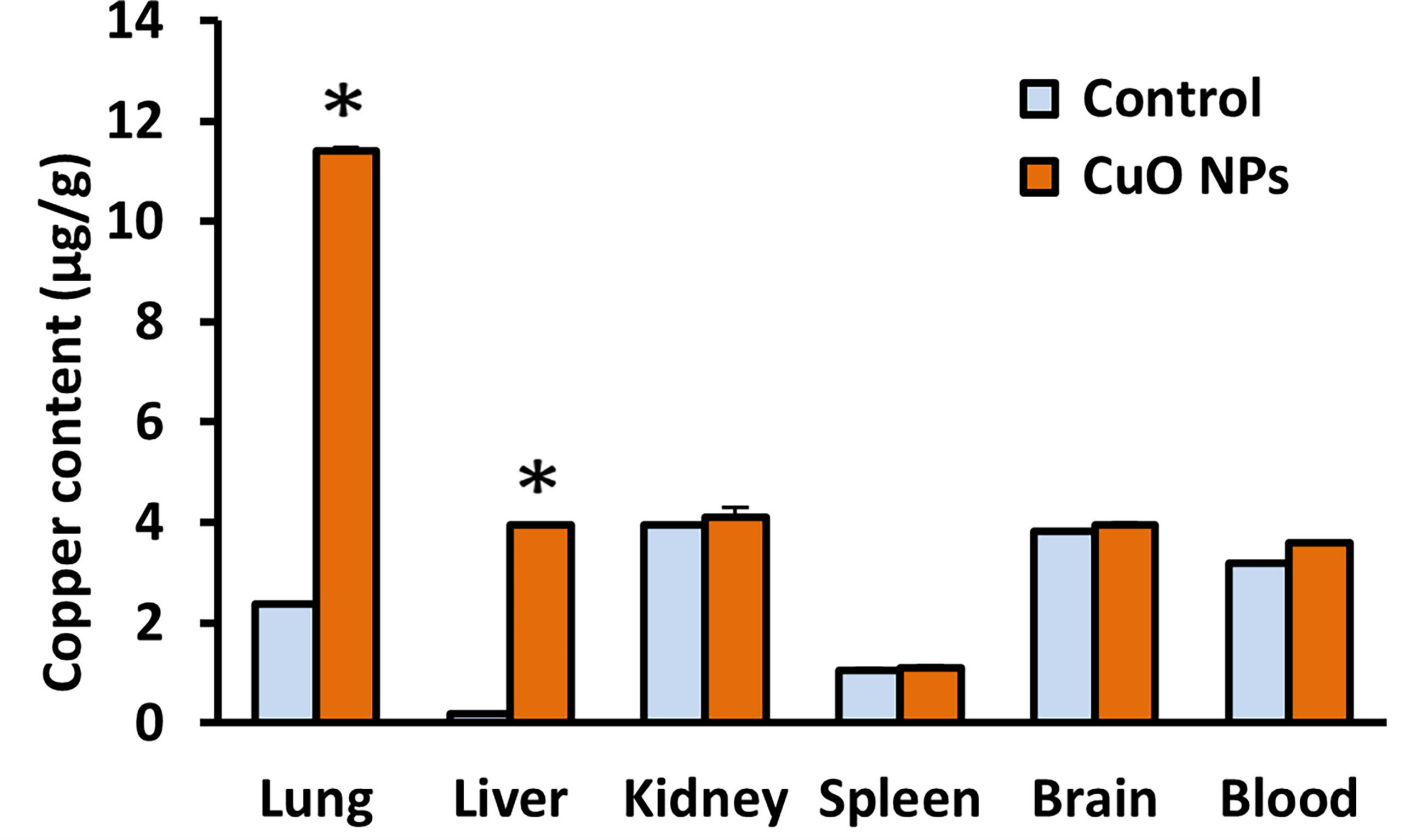
Mean content of copper in mouse organs and blood after 6-week inhalation of CuO NPs. Blood and organs were derived from mice exposed to NPs (n = 5) and controls (n = 5). Control – control group, CuO NPs – group exposed to CuO NPs. Bars indicate mean group values (mean + SD). Significance: *p < 0.05.

Cu content in five organs and blood from both exposed and control mice was analyzed at the end of the experiment. The content of Cu in lungs and liver was markedly increased after six weeks of CuO NP inhalation (**Figure 3**). The content of Cu in lungs and liver of exposed mice was respectively 4.8 times and 20 times higher in comparison to the control mice. The content of Cu in kidneys, spleen and brain, other secondary organs, and in blood was similar in exposed and control mice.

### 3.2 Phenotypic Analysis Showed an Increased Percentage of Spleen CD3^-^CD335^+^ Cells

At the end of experiment, analyses of individual cell subsets in spleen, thymus, and lymph nodes of exposed mice were performed by flow cytometry. The proportions of CD3^+^ (T-lymphocytes), CD3^+^CD4^+^ (T-helper lymphocytes), CD3^+^CD8^+^ (T-cytotoxic lymphocytes), CD3^-^CD19^+^ (B-lymphocytes) and CD3^-^CD335^+^ (natural killer (NK) cells) were analyzed. The results of phenotypic analysis are summarized in **Table 1**. The effect of CuO NPs was manifested by a significantly increased percentage of CD3^-^CD335^+^ cells in spleen of exposed mice vs. controls. The percentages of spleen CD3^+^, CD3^+^CD4^+^, CD3^+^CD8^+^, and CD3^-^CD19^+^ in exposed mice did not differ from those in control animals. In thymus, no significant effect of CuO NPs on T-cell subpopulations was observed. Similarly, there were no differences in the proportions of T-, B-lymphocytes and NK-cells in lymph nodes between the exposed and control mice.

**Table 1:** Phenotypic analysis of spleen, thymus, and lymph node cells.

Organ	Parameter	Control (Mean ± SEM)	CuO NPs (Mean ± SEM)
Spleen	CD3^+^	53.8 ± 3.9	50.6 ± 5.1
	CD3^+^CD4^+^	41.3 ± 3.6	37.4 ± 4.2
	CD3^+^CD8^+^	12.5 ± 1.5	11.1 ± 2.9
	CD3^-^CD19^+^	37.6 ± 1.8	37.8 ± 1.8
	CD3^-^CD335^+^	2.5 ± 0.1	3.3 ± 0.2*
Thymus	CD3^+^	31.6 ± 2.9	37.1 ± 6.5
	CD3^+^CD4^+^	20.4 ± 2.5	26.1 ± 4.3
	CD3^+^CD8^+^	29.7 ± 2.7	28.9 ± 3.6
Lymph nodes	CD3^+^	53.9 ± 1.8	52.8 ± 2.3
	CD3^+^CD4^+^	41.5 ± 2.1	36.8 ± 1.7
	CD3^+^CD8^+^	51.0 ± 1.8	48.2 ± 2.3
	CD3^-^CD19^+^	24.5 ± 1.6	28.7 ± 1.3
	CD3^-^CD335^+^	5.0 ± 0.8	3.8 ± 0.4

Organs were derived from mice exposed to CuO NPs for 6 weeks and controls. Cells were labelled with fluorescent monoclonal antibodies and analyzed using flow cytometer. Control – control group (n = 9), CuO NPs – group exposed to CuO NPs (n = 8). CD3^+^ – T-lymphocytes, CD3^+^CD4^+^ – T-helper lymphocytes, CD3^+^CD8^+^ – T-cytotoxic lymphocytes, CD3^-^CD19^+^ – B-lymphocytes, CD3^-^CD335^+^ – NK-cells. Results are expressed as the mean group percentage of labelled cells (mean ± SEM). Significance: *p < 0.05.

### 3.3 CuO NP Exposure Significantly Stimulated Proliferative Activity of Spleen T-Lymphocytes

**Figure 4:**
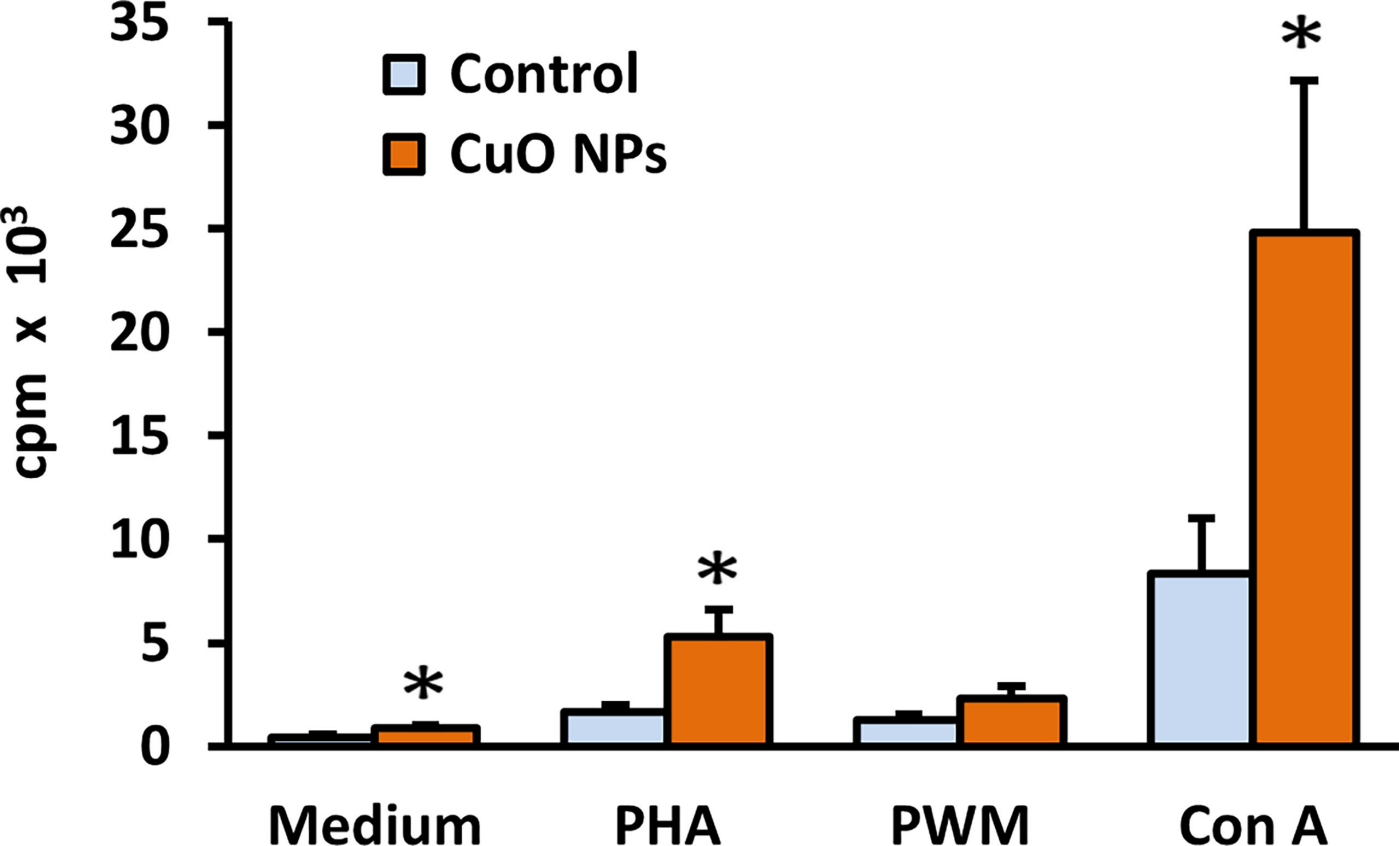
Proliferative activity of spleen lymphocytes in mice after 6-week inhalation of CuO NPs. The proliferative response of spleen T-lymphocytes and T-dependent B-cell response in mice was measured as incorporation of [^3^H]-thymidine into replicating cells. Control – control group (n = 8), CuO NPs – group exposed to CuO NPs (n = 7). Spleen cells were derived from mice and *in vitro* stimulated with mitogens: medium – unstimulated cells, PHA – phytohemagglutinin, PWM – pokeweed mitogen, Con A – concanavalin A. Results are expressed as cpm (counts per minute) in thousands per well. Bars indicate mean group values (mean + SEM). Significance: *p < 0.05.

After 6-week inhalation of CuO NPs, proliferative activity of spleen lymphocytes was examined *in vitro* after stimulation with three different mitogens (Con A, PHA, and PWM) and measured by [^3^H]-thymidine incorporation and scintillation counting. Basal proliferation of non-stimulated spleen cells was evaluated in parallel. Results are displayed in **Figure 4**. Basal proliferative activity of isolated splenocytes was significantly increased in mice exposed to CuO NPs compared to controls. Splenocytes stimulated *in vitro* with T-cell mitogens Con A and PHA showed significantly higher proliferative activity in exposed mice (297% and 314%, respectively). Stimulation of spleen B-cells with T-dependent B-cell mitogen PWM was not significantly increased in exposed mice.

### 3.4 The Inhalation of CuO NPs in Mice Resulted in Significantly Increased Levels of IL-12p70, and T-Cell Derived Cytokines IFN-γ, IL-4, and IL-5

After 6-week inhalation of CuO NPs, *in vitro* production of several key cytokines was examined using the luminescence method. The levels of IL-4, IL-5, IL-6, IFN-γ, IL-12p70, and TNF-α in spleen cell culture supernatants are summarized in **Table 2**. The inhalation of CuO NPs in mice resulted in a statistically significant increase in levels of IL-12p70, and T-cell derived cytokines IFN-γ, IL-4, and IL-5 (166%, 182%, 185%, and 1055%, respectively). Levels of TNF-α and IL-6 remained unchanged.

**Table 2:** Concentrations of cytokines in spleen cell culture supernatants in mice after 6-week inhalation of CuO NPs.

Cytokine	Control (Mean ± SEM)	CuO NPs (Mean ± SEM)
IFN-γ	51.65 ± 9.05	94.25 ± 10.35*
IL-4	59.64 ± 16.03	110.33 ± 12.92*
IL-5	3.33 ± 1.23	35.11 ± 16.36*
IL-6	6.90 ± 3.20	8.04 ± 3.04
IL-12p/70	0.41 ± 0.09	0.68 ± 0.14*
TNF-α	7.72 ± 2.04	12.26 ± 3.45

Cytokines were measured using luminescence method. Control – control group (n = 8), CuO NPs – group exposed to CuO NP NPs (n = 7). Results are expressed in ng/mL as mean group levels of cytokines (mean ± SEM). IFN-γ – Interferon-γ, IL – Interleukin, TNF-α – Tumor necrosis factor-α. Significance: *p < 0.05.

### 3.5 CuO NP Exposure Significantly Decreased Phagocytic Activity of Blood Granulocytes

**Figure 5:**
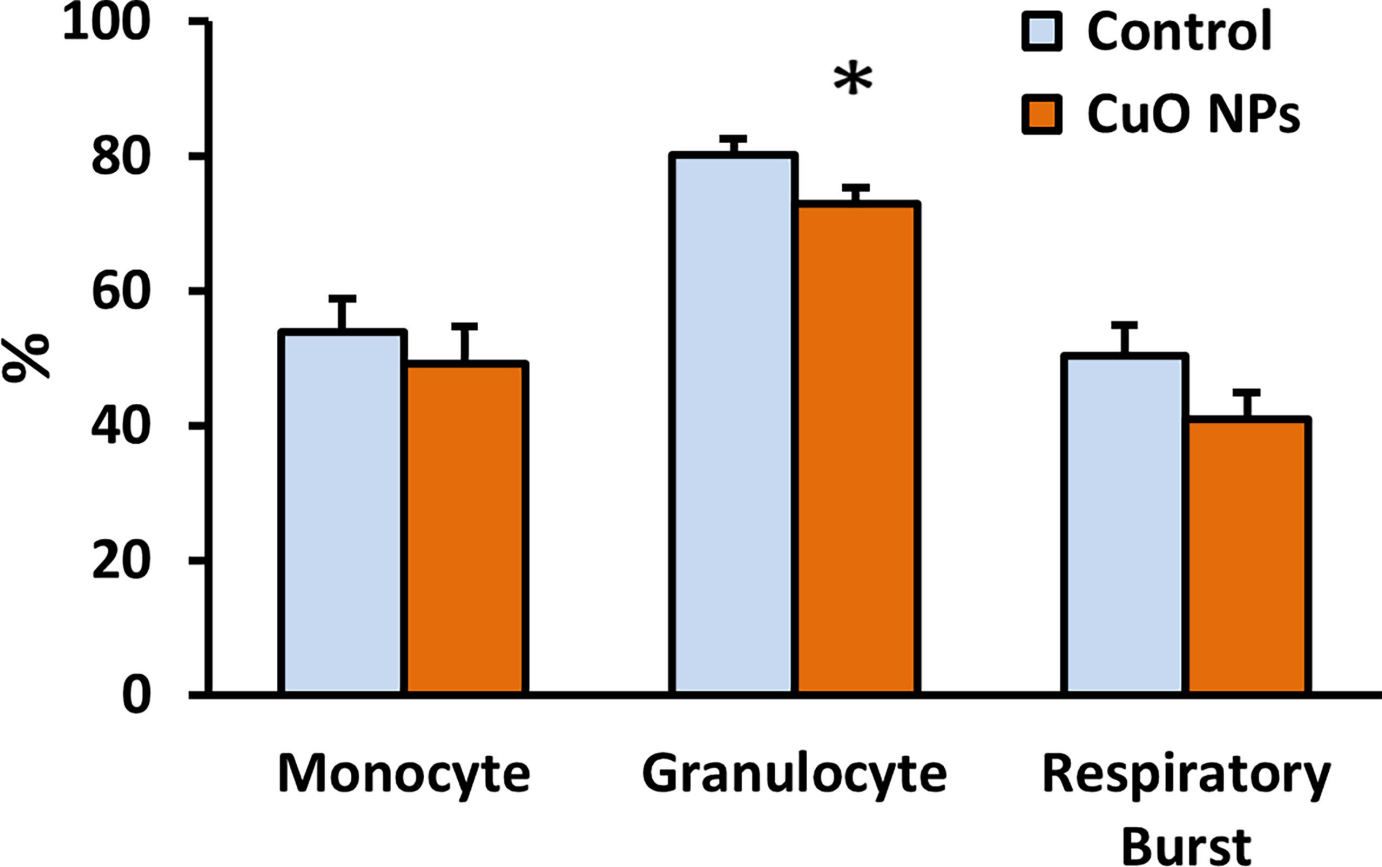
Phagocytic activity and respiratory burst of leukocytes. Phagocytic activity of monocytes and granulocytes was evaluated using ingestion of fluorescein-labelled *Staphylococcus aureus*, and the respiratory burst was monitored using hydroethidine by flow cytometry. Control – control group, CuO NPs – group exposed to CuO NPs. Blood was derived from mice exposed to CuO NPs for 6 weeks (n = 7) and controls (n = 8). Results are expressed as the percentage of phagocytic activity and respiratory burst. Bars indicate mean group activity of blood cells (mean + SEM). Significance: *p < 0.05.

The function of monocytes and granulocytes, important cells of natural immunity, was evaluated using flow cytometry. CuO NP exposure significantly decreased phagocytic activity of granulocytes (91%) in exposed mice vs. controls (**Figure 5**). The respiratory burst of leukocytes was slightly decreased in exposed mice.

### 3.6 CuO NP Inhalation Did Not Cause Significant Changes in Hematological Parameters

There were no significant differences between the CuO NP-exposed group and control group in basic hematological parameters: leukocyte count (WBC), erythrocyte count (RBC), hematocrit, hemoglobin (Hb), mean corpuscular hemoglobin, mean corpuscular hemoglobin concentration, mean cell volume, platelet count (PLT), lymphocyte count, and percentage of lymphocytes in blood (**Table 3**).

**Table 3:** Hematological parameters in mice after 6-week inhalation of CuO NPs.

Parameter	Control (Mean ± SEM)	CuO NPs (Mean ± SEM)
WBC (10^9^/L)	4.79 ± 0.56	5.20 ± 0.73
RBC (10^12^/L)	8.45 ± 0.23	8.70 ± 0.32
HGB (g/dL)	14.63 ± 0.31	14.80 ± 0.45
HCT (%)	44.86 ± 1.01	46.23 ± 1.33
MCV (fL)	53.13 ± 0.61	53.29 ± 0.55
MCH (pg)	17.38 ± 0.34	17.03 ± 0.18
MCHC (g/dL)	32.66 ± 0.35	32.01 ± 0.13
PLT (10^9^/L)	919.3 ± 57.1	986.4 ± 80.2
LYM (10^3^/μL)	3.19 ± 0.40	3.08 ± 0.45

Blood was derived from mice exposed to CuO NPs (n = 8) and controls (n = 9). WBC – Leukocyte Count, RBC – Erythrocyte Count, HGB – Hemoglobin, HCT – Hematocrit, MCV – Mean Corpuscular Volume, MCH – Mean Corpuscular Hemoglobin, MCHC – Mean Corpuscular Hemoglobin Concentration, PLT – Platelet Count, LYM – Lymphocyte Count. Control – control group, CuO NPs – group exposed to CuO NPs. Results are expressed as mean group values: mean ± SEM.

### 3.7 The Inhalation of CuO NPs Reduced Antioxidant Status in Blood

Reduced glutathione represents a main antioxidant in cells. The overall status of the antioxidant protection of the organism was determined by evaluating the concentrations of GSH and GSSG in blood samples. The experimental group exposed to CuO NPs showed a decrease (23%) in GSH concentration (96.7 ± 6.1 μmol/L) compared to the control group (125.5 ± 4.6 μmol/L); see **Table 4**. The difference was statistically significant (p = 0.007). GSSG concentrations in the exposed group (2.1 ± 0.7 μmol/L) and the control group (3.8 ± 0.7 μmol/L) were comparable (p = 0.149). Similarly, the GSH/GSSG ratio in mice exposed to CuO NPs (54.7 ± 19.3) did not differ from control mice (63.4 ± 13.4) (p = 0.700).

**Table 4:** Antioxidant status, reduced glutathione and oxidized glutathione in the blood of mice after 6-week inhalation of CuO NPs.

Parameter	Control (Mean ± SEM)	CuO NPs (Mean ± SEM)
GSH	125.5 ± 4.6	96.7 ± 6.1**
GSSG	3.8 ± 0.7	2.1 ± 0.7
GSH/GSSG	54.7 ± 19.3	63.4 ± 13.4

Blood was derived from mice exposed to CuO NPs (n = 8) and controls (n = 9). GSH – reduced glutathione, GSSG – oxidized glutathione. Results are expressed in μmol/L as mean values of GSH and GSSG (mean ± SEM). Significance: **p < 0.01.

## 4 Discussion

At the end of the 6-week inhalation experiment, Cu content in lungs, liver, spleen, kidneys, brain and blood was analyzed. The content of Cu in lungs, the organ of NP entry into body, and in liver, secondary organ, was markedly increased after six weeks of CuO NP inhalation. Analogous results were obtained after ZnO NP inhalation when comparable amounts of Zn in kidney, brain and blood and a higher content of Zn in lung and liver of exposed mice in comparison to control mice were found (37). Zn and Cu as essential elements are commonly present in various enzymes and proteins, which probably caused the enhanced natural background of Zn and Cu in organs in both control and exposed mice. Quite different results were found for the content of cadmium and lead, elements that are not naturally present in living bodies, in organs and blood of mice exposed to CdO and PbO NPs. After six weeks of CdO and PbO NP inhalation, cadmium and lead levels in lung as well as in the secondary organs (liver, kidney, spleen, brain) and blood was markedly increased in exposed mice in comparison to the control mice (38, 39).

A growing amount of evidence points to inflammation and oxidative stress playing a key role in the pathogenesis of NP-induced damage. Therefore, proinflammatory cytokines, markers of presence and function of immune cells and markers of antioxidant defense were selected in our experimental study. This study advances and extends recent findings (29) that had shown modulation of lymphocyte proliferation and production of cytokines by cells of the adaptive immune response. In our experiment, higher doses of NPs, a new functional assay and a different spectrum of cytokines were employed. To the best of the authors’ knowledge, these novel effects have not been reported to-date.

Already available *in vitro* studies have indicated possible immunotoxicity of CuO NPs mediated by cytotoxicity towards immune cells (human blood lymphocytes) (16), RAW264.7 (murine macrophage cell line) (19), or potent induction of proinflammatory cytokine TNF-α in differentiated human THP-1 cells (human monocytic leukemia cell line) (17).

Our *in vivo* study in mice assessed the effect of six-week inhalation of CuO NPs on proportions of lymphocyte subsets in lymphoid organs. Phenotypic analysis of immune cells is considered to be a sensitive indicator of possible immunotoxicity. In our study, a significant increase in proportion of NK-cells in spleen of animals exposed to 32.5 µg CuO/m^3^ for 6 weeks was observed. We suppose that the enhancement is not of biological significance. Results of T- and B-cell subset analysis published by other authors indicate a significant suppression of lymphocyte subsets: CD3+CD4+, CD3+CD8+ (40), T-cells, B-cells (29, 40) but our findings did not show any changes in spleen, thymus, and lymph nodes of mice after 6-weeks of CuO NP exposure.

In the next step, the proliferative response of lymphocytes was examined with/without mitogen stimulation. Despite the unchanged proportion of spleen lymphocyte subsets found in our study, the proliferation of splenocytes was increased in mice exposed to CuO NPs. The proliferative activity of T-lymphocytes and basal proliferative activity were significantly enhanced. Similar trends were found by Holan et al. (29) who described slight but not significant increases in reactivity of lymphocytes to mitogens Con A and lipopolysaccharide (LPS) in mice exposed to CuO NPs compared to controls. Enhanced stimulation of lymphocytes has been observed after exposure to some other engineered NPs. *In vivo* systemic administration of dextran**-**stabilized iron oxide NPs (10 mg/kg/bw) resulted in increased proliferation of spleen mitogen**-**stimulated lymphocytes in male Wistar rats (41). Intratracheal instillation with nano-TiO_2_ NPs (0.5, 4, and 32 mg/kg/bw) increased proliferation of T-cells and B-spleen cells following mitogen stimulation in Sprague Dawley rats (42). *In vitro* exposure of human peripheral blood cells to silica-activated monocyte-derived dendritic cells significantly increased T-lymphocyte proliferation (43). An increase in proliferative response to PHA was also detected in peripheral blood cells treated with Al_2_O_3_ and TiO_2_ NPs (44).

The ability of spleen cells from exposed and control mice to respond to stimulation with T-cell mitogen by production of cytokines IL-4, IL-5, IL-6, IFN-γ, IL-12p70, and TNF-α was evaluated. After 6-week inhalation of CuO NPs, significantly enhanced levels of IL-12p70 and T-cell cytokines Th1 IFN-γ as well as Th2 IL-4 and IL-5 were found in spleen cell supernatants of exposed mice vs. controls.

Similarly to our findings, Zhou et al. (40) reported significantly increased levels of cytokines IFN-γ, TNF-α, IL-4, IL-6, MIP-1α, MCP-1, MIF, IL-1, and IL-2 in nano-Cu groups in orally treated rats for 28 days. An experiment conducted with chemically identical NPs and the same route and time of exposure in mice published by Holan et al. (29) found slightly decreased production of IFN-γ, IL-2, IL-10, and significantly decreased IL-17. In accordance with our study, production of IL-6 was comparable to that observed in controls. Several other nanomaterials were found to induce this type of inflammation. Exposure of mice to single-walled carbon nanotubes elevated the levels of IL-12 and IL-6 in animal blood (45). Increased levels of TNF-α, IL-6, and IL-1β were also detected in the sputum and serum collected from workers exposed to multi-walled carbon nanotubes (46).

Significantly elevated levels of IL-4 and IL-5 indicated the activation of Th2-cells, mast cells, eosinophils, and basophils. IL-4 and IL-5 along with other Th2-cytokines are involved in the airway inflammation observed in the lungs of patients with allergic asthma. Increased levels of IL-4 and IL-5 were found in the sputum and serum of workers exposed to multi-wall carbon nanotubes (46). Patients with silicosis also had increased levels of IL-4, IL-5 IL-10, and IL-13 in their serum (47).

Overall, our results are in line with the opinion of Ma (48) on a potential mechanism for the development of NP-induced damage. The author supposes that, despite large variations in particles, the early response to inhaled particles entails a barrier immune response dominated by type 1 inflammation (phagocytosis by M1 macrophages and recruitment of neutrophils) fueled by Th1 and proinflammatory cytokines. Acute inflammation is immediately followed by resolution and tissue repair and type 2 cytokines and cells including M2 macrophages and Th2 lymphocytes.

The phagocytic activity of blood granulocytes in our study was significantly suppressed. Published papers dedicated to the effect of Cu-nanoforms on function of phagocytes are rare. A study done by Arancibia et al. (49) found that intraperitoneally administered Cu NPs (300 µg/mouse; 30-50 nm) in mice induced significant recruitment and internalization by peritoneal macrophages inducing cell death. Ray et al. (50) observed a decrease in phagocytic response in mussel hemocytes after exposure to CuO NPs.

The slightly suppressed respiratory burst of leukocytes found in our study is consistent with findings of decreased nitric oxide generation recorded in coelomocytes of earthworms exposed to 1000 mg/kg of Cu NPs (51) or decreased nitric oxide generation found in mussel hemocytes exposed to 0.5, 1 and 5 mg CuO NPs/L (50). A similar effect was shown *in vitro*, in LPS-stimulated primary macrophages. Cells treated with Cu NPs inhibited the production of nitric oxide in a dose-dependent manner (49).

The role of GSH in the defense of organism after exposure to CuO NPs has been published in recent *in vitro* studies (15–20) and *in vivo* studies (12, 23, 52, 53), after short-term or long-term exposure. Most of these authors reported a reduction in GSH levels in experimental conditions. The rate of reduction of GSH levels depends on the concentration of CuO NPs and exposure time (23, 53, 54).

Our results demonstrated a significant decrease in the GSH content, and a slight decrease in the GSSG content in blood of animals exposed to CuO NPs. These results are in agreement with other authors. Anreddy (23) reported a significant decrease in GSH levels in rats after oral 14-day administration of various concentrations of CuO NPs. Similarly, a decrease in GSH levels with increasing duration of exposure and increasing dose of CuO NPs was reported in human skin epidermal cells (15).

In contrast, higher GSH levels were reported in CuO NP-exposed salmon cells in comparison with control cells, but GSH levels decreased with increasing levels of CuO NPs in this study (54). Exposure to CuO NPs generates decreasing GSH levels, but this decline should not correspond directly to an increase in GSSG levels. Some GSH molecules may be used for the enzyme glutathione transferase in reaction forming conjugates of GSH with xenobiotics during exposure in the process of detoxification (55, 56).

The reduced form of glutathione plays an important role as antioxidant, preserving proteins and enzyme integrity (53), and can affect the antioxidant defense of organisms against various xenobiotics, including metal NPs, as confirmed by numerous studies. The harmful effect of CuO NPs is manifested in the damage of the defense mechanisms of organisms by reducing GSH levels and increasing production of ROS. The adverse effects based on the basic biochemical pathways may cause DNA damage, epigenetic changes, and apoptosis (11, 57, 58).

## 5 Conclusion

With the rapid development of the emerging field of nanotechnology, CuO nano forms became popular and widely used. Therefore, better understanding of their biological impacts following exposure is critical for assessment of potential risks to human health and the environment. Our study examined the effect of 6-week inhalation exposure to CuO NPs on possible modulation of immune/inflammatory response and antioxidant defense in mice. Innate immunity was affected by impaired phagocytic activity of granulocytes. Stimulation of adaptive immunity was indicated by activation of proliferation and secretion function of lymphocytes. Inhalation of CuO NPs caused a significant increase in the basal proliferative activity of splenocytes and the proliferative response of T-lymphocytes after mitogenic stimulation. CuO NPs significantly induced the production of IL-12p70, Th1 cytokine IFN-γ, and Th2 cytokines IL-4 and IL-5. Levels of TNF-α and IL-6 were not significantly increased. The decrease in blood antioxidant capacity indicates the severity of the detected oxidative stress. Further research is needed to reveal whether long-term exposures to CuO NPs can stimulate the progress to chronic inflammatory and immune responses leading to pulmonary/pleural chronic inflammation, fibrosis, and malignancy.

## Data Availability Statement

The datasets generated and analyzed during the current study are available from the corresponding authors on reasonable request.

## Ethics Statement

The animal study was reviewed and approved by Animal Ethics Committee of Institute of Analytical Chemistry of the Czech Academy of Sciences, Brno, Czech Republic.

## Author Contributions

JT, VM, IU, AL, MLM, MiB, and PM contributed to the writing of the manuscript. AL, MLM, ER, RA, ZK, MiS, MH, and MaS conducted the autopsy, sampling, and hematology and immune assays. MLM and LW did statistical analysis. MiB performed blood sampling, measurement, and evaluation of antioxidants. ZV, PM, PC, KK, LA, LV, and MaB developed the study concept, design of the study, performed inhalation experiment and measured NPs. JD performed TEM analysis. PP and VT measured radioactivity of tritium and together with MD, NL and DK critically revised the final manuscript. All authors gave their approval to the final draft prior to publication.

## Funding

This article was created by the realization of the project “Center of excellence of environmental health”, ITMS No. 26240120033, based on the supporting Operational Research and Development Program financed from the European Regional Development Fund. The work was supported by the European Commission 7^th^ FP (QualityNano project INFRA-2010-1.1.31, No. 214547-2); the Czech Science Foundation under grants No. P503/12/G147 and P503/20/02203S; project PROGRES Q25/COOPERATIO/LF1 Charles University in Prague; EEA and Norwegian Financial Mechanisms and the state budget of the Slovak Republic (Project SK0020); RECETOX research infrastructure (the Czech MEYS: LM2018121); CETOCOEN PLUS (the Czech MEYS: CZ.02.1.01/0.0/0.0/15_003/0000469) and CETOCOEN Excellence project (No 857560 and CZ.02.1.01/0.0/0.0/17_043/0009632).

## Conflict of Interest

The authors declare that the research was conducted in the absence of any commercial or financial relationships that could be construed as a potential conflict of interest.

## Publisher’s Note

All claims expressed in this article are solely those of the authors and do not necessarily represent those of their affiliated organizations, or those of the publisher, the editors and the reviewers. Any product that may be evaluated in this article, or claim that may be made by its manufacturer, is not guaranteed or endorsed by the publisher.

## Abbreviations

Cu, copper; CD, cluster of differentiation; CuO, copper oxide; CuO NPs, copper oxide nanoparticles; Con A, concanavalin A; GSH, reduced form of glutathione; GSSG, oxidized form of glutathione; IFN-γ, interferon-γ; IL, interleukin; NK-cells, natural killer cells; NP, nanoparticle; PHA, phytohemagglutinin; PWM, pokeweed mitogen; ROS, reactive oxygen species; TNF-α, tumor necrosis factor-α
